# Consumer knowledge, preference, and perceived quality of dried tomato products in Ghana

**DOI:** 10.1002/fsn3.439

**Published:** 2016-11-03

**Authors:** Mavis Owureku‐Asare, R. P. Kingsly Ambrose, Ibok Oduro, Charles Tortoe, Firibu K. Saalia

**Affiliations:** ^1^ Department of Agricultural and Biological EngineeringPurdue UniversityWest LafayetteINUSA; ^2^ Department of Food ScienceKwame Nkrumah University of Science and TechnologyKumasiGhana; ^3^ CSIR‐Food Research InstituteAccraGhana; ^4^ Department of Nutrition and Food ScienceUniversity of GhanaLegonGhana

**Keywords:** Consumer perceptions, quality, solar drying, tomato

## Abstract

Postharvest losses (PHL) are incurred in the tomato value chain in Ghana and solar drying of tomato is a promising technology for reducing the loss. However, there are concerns on the usage, functionality and sensory appeal of the dried products to consumers, compounded with the lack of information and research on dried tomato processing in Ghana. A survey was carried out by administering semistructured questionnaires to 395 randomly selected and willing respondents in the Accra Metropolis. Information was obtained on the socioeconomic profile, consumption pattern, knowledge, and acceptance of tomato processing technologies and assessment of quality attributes important to consumers. Most consumers (74%) preferred tomato powder that is conveniently packaged to retain the characteristic intense taste and the flavor using Friedman's rank mean procedure. The study indicated that consumers were more concerned about good manufacturing practices during the production of solar‐dried tomato (48.8%) rather than the quality attributes (8.6%). These findings indicate the need for safe solar drying procedures in order to increase consumer acceptability of solar‐dried tomato products in Ghana.

## 1 Introduction

Tomatoes are widely cultivated in Ghana and much of it is used locally as vegetable. Currently, it is mostly consumed in its fresh and canned states (tomato paste). Tomato is used as an ingredient in the preparation of a variety of Ghanaian staples. The high moisture content of tomatoes (above 95%) and its soft outer covering predisposes it to microbial spoilage and short shelf life, leading to high annual postharvest losses (PHL) (20–50%) of the crop (Kader, 1992). Industrial processing of tomatoes in Ghana remains very low due to a variety of reasons, such as irregular raw material supplies, limited varieties ideal for processing, poor maintenance cycles of equipment and the lack of adequate skilled labor (Robinson & Kolavalli, 2010). Consequently, alternative processing methods, such as drying of fresh, ripe tomato into dried tomato products, need to be explored to extend the shelf life as well as add value to the crop.

Even though dried tomatoes are not commonly used in the preparation of meals, compared to fresh tomato and canned tomato products, the concept of dried tomatoes is not entirely new among food processors and some consumers especially in the tomato growing areas in Northern Ghana. In this region, some farmer's sundry tomatoes for commercial purposes and to prevent it from rotting when patronage and sales of fresh tomatoes are low. Sun drying of tomato for household consumption is also practiced in this region. A product made using milled annatto seeds, corn, cola nuts, and E color series (to impart red color), is being sold as “tomato powder” in Accra. The product is highly patronized by cottage food processors and street food vendors as an inexpensive substitute for tomato paste. The existing demand for this product suggests that hygienically processed solar‐dried tomatoes of good quality with desirable characteristic color will be greatly appreciated by food processors and consumers alike.

Several methods of drying have been employed for food (Doymaz & Pala, 2002; Ekow, 2013; Kingsly, Singh, Goyal, & Sigh, 2007; Telis & Sobral, 2002), and among them solar drying of tomatoes and other vegetables has shown a great deal of promise. The technology of solar drying has been practiced for different foods for decades, and it is a promising technology for drying tomatoes even though it has rarely been used for that purpose. Drying using electric (convention) ovens is more commonly practiced, but it is also much more expensive due to the high moisture content in fresh tomatoes and the associated energy costs. In rural Ghana, and in many tropical rural communities in the world where there is poor access to the National electric grid, solar dryers can be used to dry tomatoes, and save considerably on electric energy (Afriyie, Rajakaruna, Nazha, & Forson, 2011; Belessiotis & Delyannis, 2011; Ekechukwu & Norton, 1999). There are several types of solar dryers, but the passive type, whose operation does not depend on electric energy, is more suited for tropical communities (Afriyie et al., 2011). They are the easiest and most economical to operate, and their efficiency can be improved by optimizing the dryer design to increase airflow, insolation, and drying speed for the product.

While drying tomatoes in a solar dryer may appear easy, practical, and inexpensive, the same might not be said for consumer acceptability of the dried products for food applications. Dried tomatoes have functional and quality characteristics that are different from fresh tomatoes, and product quality characteristics are very important in consumer choices. Moreover, fresh tomatoes are more traditional and culturally accepted in food applications. Cultural factors influence food choices (Rozin & Vollmecke, 1986) because of differences in both perception and preference (Prescott & Bell, 1995). Consumers’ perceptions are dynamic (Koster & Mojet, 2007) and the usage and demand of a product may depend more on the consumer's perception about the product. For traditionally or locally produced food products, consumers rarely make food choices in the absence of extrinsic factors of personal, social, and cultural significances (Paxson, 2010; Sutton, 2010; Trubek, 2008). Alphonce, Temu, and Almli (2015) showed that consumer preferences for dried fruit are affected significantly by its typical aroma intensity.

Several studies have also examined the relationship between consumer perceptions or preference and how that is incorporated into the quality characteristics of the product through the production process (Cerjak, Karolyi, & Kovacic, 2011; Chrea et al., 2011; Korzen & Lassen, 2010; Mueller & Szolnoki, 2010). These are important prerequisites for market success of a new product, especially at an early stage of their transformation into marketable products (Siegrist, 2008). Consumers are becoming more concerned about hygiene and quality of foods and are willing to pay more for the solar‐dried products (Agribusiness Development Centre, 2001). The quality of dried tomatoes when assessed by consumers may or may not influence the purchase of the product; however, consumer surveys provide information needed to manage and shift consumer expectations on new products (Siegrist, 2008). This study sought to gather information on consumer's knowledge, preferences, and product assessment which will serve as basis for characterization and formulation of dried tomato products in Ghana.

## 2 Materials and Methods

### 2.1 Methodology

A survey was carried out by administering semistructured questionnaires to randomly selected and willing respondents in the Accra Metropolis. Preliminary survey was conducted to pretest questionnaires using 25 subjects. Response gathered was used to validate and modify questionnaires used in the survey. Based on the method described by Moore and McCabe (1993), a sample size of 384 was obtained using a margin of error of 5%. This was increased to 395 subjects used in the study. Although self‐administration of questionnaire was encouraged, in situations where respondents could not fill out the questionnaire independently, field assistants were available to help them write out their responses (in a language of mutual understanding) as accurately as possible. Questions were designed to assess among other things, consumer's preference, ranking, and scoring of desirable quality attributes of dried tomatoes. Information on product preference, production quality and safety assessment, and packaging preference for dried tomatoes were provided by consumers. Information gathered on product quality attributes will serve as a baseline and guide in the production of dried tomatoes using solar drying technique.

### 2.2 Study location

The locations for sampling in the survey included all five residential classes: northern, central, southern, eastern, and western parts of Accra according to the Accra Metropolitan Development Classification of Accra (the capital of Ghana).

### 2.3 Data analysis

Data entry and analysis was done using Statistical Package for Social Sciences (SPSS version 16.0). Frequencies were generated for variables and significant associations were tested at *p *≤ .05 using chi‐square test. Information including preference of tomato products, ranking product preference, ranking of quality attributes and packaging preference for dried tomatoes were gathered from potential consumers.

## 3 Results and Discussion

### 3.1 Socioeconomic characteristics of respondents

Factors that influence food choices of consumers, demographic information including gender, age, level of education, and marital status of the respondents were analyzed in Table 1. Most often, females decide on products for cooking or for daily use in the home, women (57.2%) were therefore more willing to respond to this survey compared to males. The majority of respondents were aged below 35 years (Table 1). This indicates that the younger generation is more curious or “adventurous” and willing to participate in a survey, the findings of which could potentially have some influence on their food choices. The age of the respondents also aligns with the marital status of the respondents with majority of them being single. Of the 395 respondents, 390 were Ghanaians and an overwhelming majority of the respondents (95.4%) had some form of formal education. Aside from students (27.8%) and a few (6.6%) who were not engaged in any gainful employment, most respondents (65.6%) were engaged in one form of income generating activity and had purchasing power for buying commercial and/or novel food products.

**Table 1:** Socioeconomic characterization of respondents in a consumer survey of dried tomato products in Accra metropolis, Ghana

Demographic variable	Number of respondents	Percentage (%)
Gender		
Male	169	42.8
Female	226	57.2
Total	395	100.0
Age		
<25	183	46.3
26–35	107	27.1
36–45	62	15.7
46–55	32	8.1
56+	11	2.8
Total	395	100
Region of respondents by birth		
Greater Accra region	82	21.0
Central region	63	16.2
Western region	20	5.1
Eastern region	68	17.4
Brong Ahafo region	10	2.6
Volta region	68	17.4
Northern region	15	3.8
Upper east region	5	1.3
Upper west region	4	1.0
Ashanti region	55	14.1
Total	390	100.0
Marital status		
Married	114	28.9
Single	259	65.6
Divorced/separated	12	3.0
Widowed	10	2.5
Total	395	100.0
Highest educational status		
None	18	4.6
Primary	8	2.0
Junior high school/O level	62	15.7
Senior high school/A level	101	25.6
Tertiary	206	52.2
Total	395	100.0
Main occupation		
Unemployed	26	6.6
Self employed	134	33.9
Private sector	89	22.5
Civil/public servant	26	6.6
Student	110	27.8
Apprentice	10	2.5
Total	395	100

### 3.2 Consumer preference and patronage of tomato products

The respondents had fairly uniform and near unanimous perceptions and opinions in their choice of tomato products. Majority of them (93%) like tomato products either extremely or moderately (Table 2). While students generally showed moderate preference for tomato products, other respondents irrespective of occupation showed extreme preference for them (Table 2). Almost half (47.6%) of the respondents sampled will buy dried tomato products from the open market rather than from a shop or supermarket. The cost of food items is usually cheaper when bought from local markets than shops and supermarkets. To drive demand for dried tomato products, the quality should be appropriate for the local market. Occupation or socioeconomic standing did not significantly influence the respondents’ choice for the open market over supermarkets (Table 2). The quality of dried tomatoes in the local market should be monitored, assessed, and improved because it is the most preferred point of sale patronized by consumers (Table 2). Only 17.2% of consumers will prefer to process dried tomatoes themselves.

**Table 2:** Association between occupation and preference of dried tomato products

Description	Occupation						*χ* ^2^	df	*p*‐value
How much do you like tomato or tomato products?	Unemployed	Self‐employed	Private sector	Civil/ public servant	Student	Apprentice			
Extremely	53.8	70.9	62.9	64.0	40.0	100	38.712	12	.01
Moderately	34.6	21.6	29.2	32.0	53.6	0			
Slightly	11.5	7.5	7.9	4.0	6.4	0			
How would you prefer to obtain dried tomato?									
Prepare yourself	11.8	23.2	14.1	6.2	15.2	25.0	15.314	18	.64
Open market	52.9	49.5	43.8	43.8	48.5	37.5			
Shop	23.5	12.6	15.6	6.2	13.6	12.5			
Supermarket	11.8	14.7	26.6	43.8	22.7	25.0			

χ^2^, chi‐square; df, degree of freedom; and significance at *p *≤* *.05.

Consumer rankings for tomato products are presented in Table 3. The data show that tomato products may be divided into two significantly different groups based on preference rankings: familiar tomato products and nonfamiliar products. Fresh tomatoes and canned tomato products are quite familiar to most consumers and were highly ranked with no significant differences (*p *>* *.05) in preference for one product over the other. On the other hand, cut dried tomatoes and powdered tomatoes are less familiar to consumers, were ranked extremely low, with no significant *(p > *.05) differences between them. Tomato paste and fresh tomatoes are used in a wide variety of soups, sauces, and stews mainly to impart flavors and color (Naika, de Jeude, de Goffau, Hilmi, & van Dam, 1989; Aggey, Amoah, and Banir, 2007) because they are readily available in the market. Aggey et al. (2007) reported that at least 7 in 10 households use tomato paste in preparing their meals during lean tomato season. On the other hand, consumption of dried tomatoes and tomato powder appears to be very low. Adimabuno (2010) observed that because the processing of sun or solar drying of tomatoes is tedious, laborious farmers prefer to sell tomatoes fresh than in the dried form. The preparation step for dried tomatoes involves washing, cutting, parboiling (optional) of tomatoes before drying in the open sun. The preparation steps can be modified by introducing mechanical cutters for size reduction and introduction of solar dryers that will reduce contamination of the dried products. This could help promote drying of tomatoes as way of absorbing excess tomato supply at the peak of the production season and also make the products more appealing to consumers.

**Table 3:** Consumer preference ranking of tomato products in Accra, Ghana

Tomato product	Rank (mean ± SD)
Fresh tomato	3.89^a^ ± 0.810
Canned tomato	2.77^ab^ ± 0.669
Cut dried tomato	1.05^c^ ± 0.588
Tomato powder	1.02^c^ ± 0.718

^*^Maximum Friedman’s rank mean is 4, where 1 is least preferred and 4 is most preferred, Values with the different alphabets at superscript along the column are significantly different *p* = .05.

Fresh tomatoes of high quality are red in color, firm texture, and good in taste and flavor. Although the fruit comes in different colors, such as red, pink, yellow, and orange, the characteristic red color is the most desired (Latapi & Barett, 2006; Yahia & Brecht, 2012). The data in Table 4 show that consumers buy fresh tomatoes based on the color (Friedman’s rank mean = 3.17 of 5) and not the flavor (rank mean = 2.64 of 5). These data suggest that color, functionality, and taste were the most critical attributes of fresh tomato consumers seek. Attributes of dried tomatoes are also very important to consumers as revealed by the significant differences observed in their rank means (*p *< .05). Even though the characteristic red color was the most desirable quality attribute associated with fresh tomatoes, taste and flavor were ranked as the most desirable attributes for dried tomato products. Flavor and color mainly affects commercialization of tomatoes (León‐Sánchez et al., 2009), thus these quality attributes must be enhanced.

**Table 4:** Assessment of consumer desirable attributes for fresh and dried tomatoes

Fresh tomato attributes	Rank (mean ± SD)	Dried tomato attributes	Rank (mean ± SD)
Color	3.17^a^ ± 1.27	Color	1.05^cd^ ± 1.44
Functionality	3.1^a^ ± 1.24	Functionality	2.5^b^ ± 2.01
Taste	3.04^b^ ± 1.38	Taste	4.3^a^ ± 1.62
Texture	2.94^b^ ± 1.36	Texture	1.9^bc^ ± 1.02
Flavor	2.64^c^ ± 1.41	Flavor	2.74^ab^ ± 1.33

Values with the different alphabets at superscript along the column are significantly different *p *=* *.05.

Taste and aroma constituents which influence the flavor of tomatoes, are mainly affected by interactions between sugars and acids (citric and malic) and are responsible for sweetness, sourness and overall flavor intensity in tomatoes (Malundo, Shewfelt, & Scott, 1995); Stevens Kader, Albright‐Holton, & Algani, 1977, Stevens, Kader, & Albright, 1979). Consumers patronage of tomato products presented in Table 5 indicates that majority of the respondents (69.5%) consumed tomatoes at least once daily, while only 4.1% consumed at least once a week. Most individuals consume tomatoes every week and all year round, irrespective of season, so the development of solar‐dried products could make dried products available for consumers. The study also revealed that most of the respondents (83.5%) do not use alternative ingredients as substitute to tomato products during the lean season of tomatoes.

**Table 5:** Consumption pattern of tomato products

Variable	Number of respondents	Percentage (%)
How often do you consume foods containing tomato products (in a week)?		
Very often (every day)	275	69.6
Often (at least 3–6 days in a week)	104	26.3
Not that often (at least 1 day in a week)	16	4.1
Total	395	100.0
What alternative ingredients do you use when fresh tomatoes are not available or in season?		
No alternative ingredients	330	83.5
Alternative ingredients	65	16.5
Total	395	100
Have you patronized dried tomato products before?		
Yes	13	3.3
No	382	96.7
Total	395	100

### 3.3 Consumer patronage of fresh and processed tomato products

**Figure 1:**
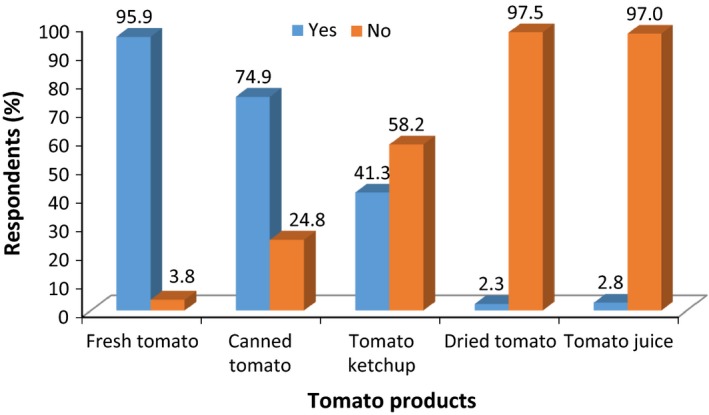
Consumer patronage of tomato products in Ghana (*n *=* *395)

Figure 1 shows high consumer patronage for fresh tomatoes (95.9%) because of its utilization in most Ghanaian sauces (Tambo & Gbemu, 2010). Canned tomatoes are the second most patronized (74.9%) tomato product with the least being dried tomatoes (2.3%) and tomato juice (2.8%). The seemingly low availability of good‐quality dried tomato products in the market could be a reason for the low patronage of the product. The introduction of improved solar drying technologies that could retain some nutrients of fresh tomatoes with improved reconstitution characteristics comparable to tomato paste could enhance consumer patronage of dried tomato products.

More than half of consumers (68%) sampled are willing to patronize dried tomatoes should the quality be improved and made readily available in the market (Table 6). Majority (74%) of the respondents prefer dried tomato in the powdered form than cut (halves) as this was least preferred (8%) by respondents. Currently the low patronage of tomato powder among Ghanaian consumers could be due to the lack of knowledge on the potential uses and functionality of dried tomatoes for preparing local dishes. Consumers would patronize dried tomatoes because of convenience (65.6%) and if readily available for purchase (48.6%). Of consumers, 26% also indicated that they would mostly produce dried tomatoes by sun drying than exploring the use of solar dryers.

**Table 6:** Consumer preference for dried tomato products

Variable	Respondents (%)	
	Yes	No
Will you consume dried tomato products?	32	68
What is your preference for dried tomato products?		
Half dried	8	92
Diced dried	18	82
Tomato powder	74	26
What is your reason for patronizing dried tomato?		
Convenience	65.6	34.4
Availability	48.6	51.4
Suitability	35.1	64.9
Cost	33.3	66.7
Other	6.3	93.7
What mode of drying would you use for tomato?		
Sun drying	46	54
Solar drying	26	74
Oven drying	28	72

### 3.4 Knowledge of economics of the tomato distribution system

Consumer knowledge can be identified in two main components: familiarity of the product and its functionality or performance as an ingredient in a related product (Alba, Wesley Hutchinson, & Lynch, 1991). About 91% of consumers were not aware of the availability or sale of dried tomato products in the market (Table 7), though the processing of dried tomato products is not entirely new in the country. Even though many of the respondents were not aware of the availability or the sale of dry tomato products in the market, this does not imply that they had no perception or knowledge of the product. As indicated in the study, they would patronize the product if products were readily available. Dried tomatoes are processed in some parts of the country, but are not common or easy to find in shops or in the open market. They are usually processed on the household scale in some tomato producing communities.

**Table 7:** Consumer knowledge of tomato economics in Accra Metropolis

	Number of respondents	Percentage (%)
Are you aware of the fluctuations in the price of tomatoes?		
Yes	327	82.8
No	68	17.2
Total	395	100
Are you aware of the postharvest losses of tomato?		
Yes	297	75.2
No	98	24.8
Total	395	100
To what extent do postharvest losses contribute to price fluctuations of tomato?		
High	151	50.8
Moderate	114	38.4
Low	32	10.8
None	98	24.81
Total	395	100.0
Are you aware of the production and sale of tomato powder in some markets in Ghana?		
Yes	36	9.2
No	359	90.8
Total	395	100

Tomato growers lose more than 40% of their produce before it reaches the final consumer due to poor postharvest handling (Gustavsson, Cederberg, Sonesson, Van Otterdijk, & Meybeck, 2011). In Ghana, at the height of the harvest season, farmers may lose about 20–50% of produce due to the lack of adequate processing facilities which results in severe price fluctuations during the year (Kader, 1992). Majority of the consumers (82.8%) were aware of price fluctuations of tomatoes and 75.2% aware of the PHL of tomatoes (Table 7). About half of respondents (50.8%) believe that PHL highly contribute to price fluctuations in prices of tomato and there is the need for introducing processing technologies to reduce PHL and regulate or stabilize tomato prices.

### 3.5 Consumer perceptions of dried tomatoes quality

Although no significant (*p > *.05) association was established between educational background and the options for mode of drying tomato (χ^2^=10.434, df=8, *p *=* *.236) (Table 8), solar drying was highly embraced by respondents with tertiary (39.7%) and secondary (27.9%) education. The mode of drying, rate of drying and reactions occurring during drying can affect the quality of the dried products (Sabarez, 2008). When tomatoes are dried in a controlled environment, there is a low likelihood of contamination by pests and other extraneous materials such as dust. In comparison to open sun drying, the drying time for solar dryers can be reduced by about 65%, improving the hygienic quality, facilitating the removal of moisture, and preventing the products from environmental factors such as rain, dust, and insects (Mechlouch et al., 2012). The proposed improved cabinet solar dryer is portable and can be used at the household level by the consumers.

**Table 8:** Association between education and processing quality of dried tomato products

Description	Highest educational level achieved (%)					*χ* ^2^	df	*p*
What method will you use to prepare dried tomato?	None	Primary	Junior high school	Senior high School	Tertiary			
Open sun drying	54.5	50.0	41.3	52.1	44.1	10.434	8	.236
Solar drying	27.3	50.0	32.6	26.0	21.3			
Oven drying	18.2	0	26.1	21.9	34.6			
Do you have concerns about the quality of dried tomato products?								
Yes	50.0	50.0	41.9	52.5	49	1.733	4	.785
No	50.0	50.0	58.1	47.5	51			

χ^2^, chi‐square; df, degree of freedom; and significance at *p *≤* *.05.

**Figure 2:**
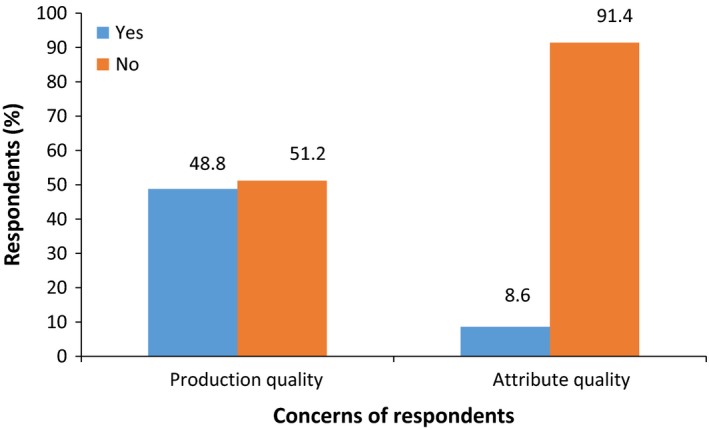
Consumer concerns on quality of dried tomato products (*n *=* *395)

**Figure 3:**
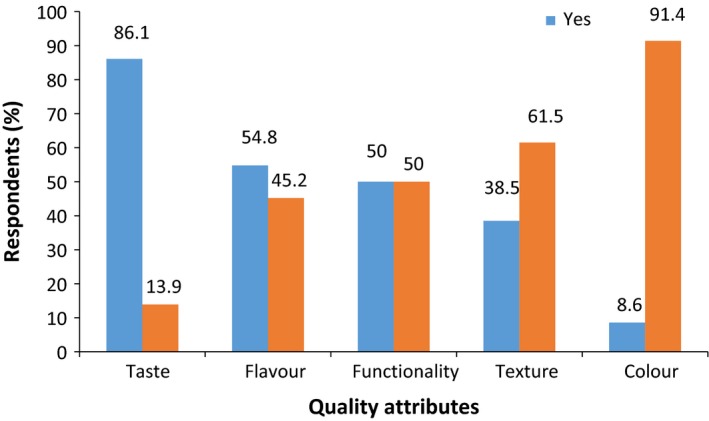
Consumer concerns about attribute quality of dried tomato products (*n *=* *84)

**Figure 4:**
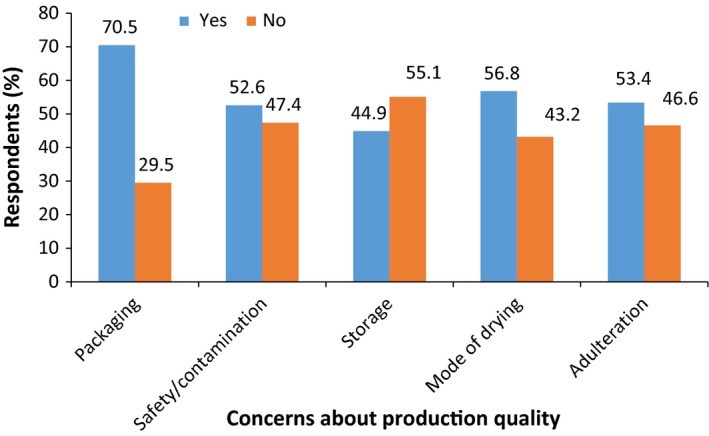
Consumer concerns about production quality of dried tomato products (*n *=* *154)

Respondents concern about the quality of dried tomatoes was categorized into two groups: concerns about the quality during production and attribute quality (Figure 2). Concerns of respondents are more often about the production quality rather than quality attributes of dried tomato products (Figure 2). Fresh tomatoes are commonly selected by consumers on the basis of appearance, with color (Latapi and Barett, 2006) being the most important quality attribute, but repeated purchase will depend on other quality attributes such as taste, texture, nutritional value, and food safety (Yahia & Brecht, 2012). Fresh tomatoes are important sources of lycopene, vitamin C, and are valued for their color and flavor, on the other hand, dried tomatoes are rich in flavor, minerals, and fiber (Kingsly et al., 2007). Majority of the respondents (86.1%) are more concerned about changes in the characteristic flavor and taste of tomato (Figure 3) and this must be preserved to a greater extent by the choice of the drying process. Most of the respondents (70.5%) indicated that they have concerns about packaging of products (Figure 4). They indicated appropriate packaging such as metalized polyethylene bags, low‐ and high‐density polyethylene bags, and polyethylene terephthalate (PET) that will preserve and extend the shelf life of dried tomatoes. The mode of drying tomato and issues of adulterating of the products were of major concern to consumers. Preferred packaging materials indicated by consumers will be used in packaging and shelf‐life studies of tomato powder and the appropriate packaging material that will maintain its quality will be selected for the final product.

## 4 Conclusions

The survey gathered vital information from consumers which are critical for developing solar‐dried tomato products in Ghana. A majority of respondents did not know about the production and availability or the sale of dried tomato products in the market, but are mostly aware of the high PHL incurred in the tomato value chain and its effect on the fluctuation in prices of tomato. Respondents are willing to purchase tomato powder. As such there is a promising market for the commercial production, marketing, and patronage of tomato powder which are conveniently packaged to retain the characteristic intense tomato taste and flavor desirable to consumers. Respondents were mostly concerned about production quality rather than attribute quality of dried tomato. The baseline information gathered from consumers on the quality characteristic desirable for a dried tomato product is helpful in the formulation and processing of solar‐dried tomatoes as a way to minimize PHL and enhance tomato processing in Ghana.

## Conflict of Interest

None declared.
